# Experiences With Video Consultations in Specialized Palliative Home-Care: Qualitative Study of Patient and Relative Perspectives

**DOI:** 10.2196/10208

**Published:** 2019-03-21

**Authors:** Karen Frydenrejn Funderskov, Mette Raunkiær, Dorthe Boe Danbjørg, Ann-Dorthe Zwisler, Lene Munk, Mia Jess, Karin Brochstedt Dieperink

**Affiliations:** 1 REHPA - Danish Knowledge Centre for Rehabilitation and Palliative Care University of Southern Denmark Nyborg Denmark; 2 Centre for Innovative Medical Technology, CIMT Department of Clinical Research University of Southern Denmark, Odense University Hospital Odense Denmark; 3 Quality of Life Research Center Department of Haematology Odense University Hospital Odense Denmark; 4 Department of Oncology Odense University Hospital Odense Denmark

**Keywords:** palliative care, relatives, telemedicine, qualitative research

## Abstract

**Background:**

The work of specialized palliative care (SPC) teams is often challenged by substantial amounts of time spent driving to and from patients’ homes and long distances between the patients and the hospitals.

**Objective:**

Video consultations may be a solution for real-time SPC at home. The aim of this study was to explore the use of video consultations, experienced by patients and their relatives, as part of SPC at home.

**Methods:**

This explorative and qualitative study included palliative care patients in different stages and relatives to use video consultations as a part of their SPC between October 2016 and March 2017. Data collection took place in the patients’ homes and consisted of participant observations followed by semistructured interviews. Inclusion criteria consisted of patients with the need for SPC, aged more than 18 years, who agreed to participate, and relatives wanting to participate in the video consultations. Data were analyzed with Giorgi’s descriptive phenomenological methodology.

**Results:**

A number of patients (n=11) and relatives (n=3) were included and, in total, 86 video consultations were conducted. Patients participating varied in time from 1 month to 6 months, and the number of video consultations per patient varied from 3 to 18.

The use of video consultations led to a situation where patients, despite life-threatening illnesses and technical difficulties, took an active role. In addition, relatives were able to join on equal terms, which increased active involvement. The patients had different opinions on when to initiate the use of video consultations in SPC; it was experienced as optional at the initiating stage as well as the final stage of illness. If the video consultations included multiple participants from the SPC team, the use of video consultations could be difficult to complete without interruptions.

**Conclusions:**

Video consultations in SPC for home-based patients are feasible and facilitate a strengthened involvement and communication between patients, relatives, and SPC team members.

## Introduction

### Background

Palliative care, according to the World Health Organization (WHO), aims to improve quality of life for patients and their families facing physical, psychosocial, or spiritual problems associated with life-threatening illnesses [1]. Specialized palliative care (SPC) is recommended for people suffering from life-threatening illnesses with complex palliative care needs. In 2015, cancer was the most common cause of death (n=15,953) in Denmark [2]. Internationally and in Denmark, patients with cancer represent the majority of patients receiving SPC [3,4]. In Denmark, from January 2010 to 2015, 9782 cancer patients, who died within this period, were referred to SPC [3].

In Denmark, as in several other European countries, SPC is provided at hospices, special palliative wards at hospitals, or in the patient’s own home by multidisciplinary SPC teams in collaboration with community nurses and general practitioners [5]. The work is often made difficult owing to long distances and the amount of time SPC teams spend driving to the patients’ homes [6,7]. Many consultations are conducted via telephone; however, the lack of nonverbal communication is a disadvantage as health care professionals overlook signs of treatable conditions or deteriorations [7]. Furthermore, relatives or community nurses usually take over telephone consultations when the patient’s illness progresses, which makes it difficult to maintain the relation between the SPC team and patient [7].

### Objectives

The increasing use of telehealth in palliative care is generally accepted, given that increased home care is seen as the preferred option by both patients and health care professionals [8-10]. The first randomized controlled trial measuring the effectiveness of weekly SPC teleconsultations was conducted in Holland based on patient-experienced symptom burden [11]. The study concluded that adding weekly teleconsultations compared with usual palliative care led to higher reported symptom scores among patients with advanced cancer but also a high degree of satisfaction with telemedicine. Hoeck et al advice future research to focus on the potential impact of technology as on the patient’s sense of well-being and appropriate timing for teleconsultations [11].

A high level of user satisfaction was also reported in a systematic literature review from Chile, and the number of hospital admissions decreased by 66% and number of bed days by 77% after introducing video phone devices and text messaging in palliative care [12,13]. However, Denmark has yet to initiate the use of video consultations in the palliative care setting as only 1 Danish qualitative study has been conducted, where 17 health care professionals discussed the opportunities found in using telemedicine without trying it but expressed a preference for face-to-face contact for optimal communication with the patients [7].

Ahead of this study, a literature review of qualitative studies was conducted to explore how video consultations work between patients and their relatives, and health care professionals in SPC. In total, 8 studies were found. There is an increasing use of telemedicine in palliative care, and it seems to be effective in the care of patients with palliative needs [6,14]. An American study showed that pain management, coordination of care, and technical issues using technology were main topics being discussed between 4 interdisciplinary hospice teams and 12 caregivers for hospice patients during videoconferencing [15]. An early introduction leads to the most benefit; however, the technologies must be reliable, mobile, and easy to use [9,16]. The use of video favors the nonverbal communication of emotions, body language, and facial expressions of suffering and happiness [17]. In total, 18 patients receiving palliative care emphasized that being able to see one another’s facial expression and detect situational contexts allows them to be absorbed in a digital connectedness [18]. In the care of complex patients, teleconsultations allowed the health care professionals in primary and in specialized care teams to support each other when a concentrated direct interaction was performed [8].

However, the above studies did not explore the opportunities for patients and their relatives in the direct involvement in SPC with the health care professionals. Therefore, the purpose of this study was to clarify if, when, and how the use of video consultations is feasible in an SPC setting and to explore how patients in their own homes and with complex palliative needs and their relatives experience the use of video consultations.

## Methods

### Overview

This study was carried out as an explorative, qualitative study with the purpose of exploring the experiences connected to video consultations. A descriptive phenomenological approach and participant observations followed by semistructured interviews were used to investigate patient and relative experiences in the use of video consultations in SPC at home [19].

### Video Consultation Intervention

This study was conducted from October 2016 to February 2017 and initiated by an SPC team at the Department of Oncology at a university hospital.

**Figure 1:**
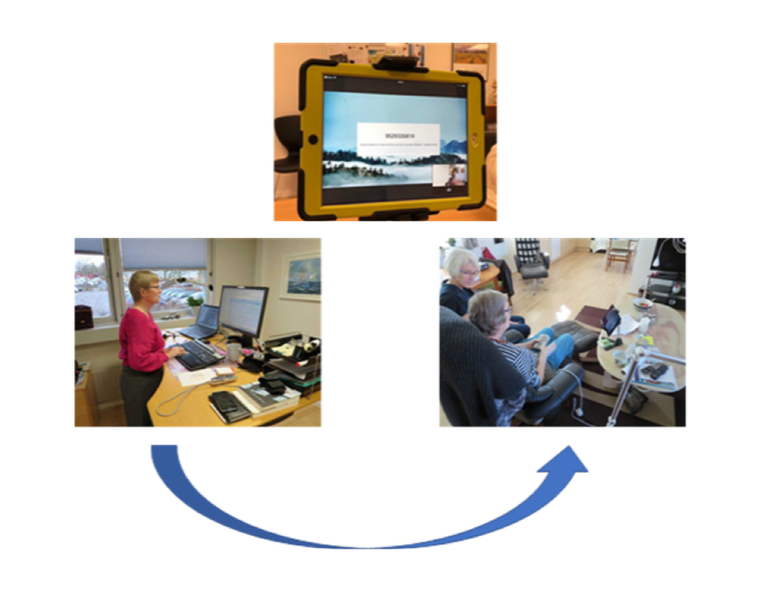
Tablet device used in video consultations in specialized palliative care.

The included patients were consulted in their homes during initial home visits by an SPC team, who asked for consent to participate. The patients and their families were primarily in telephone contact with the SPC team nurse, who then consulted the SPC team physician, when necessary. Approximately once a week, a video consultation was set by the SPC team nurse and the patient. The technical application was a tablet used for one-way calls between patients, relatives, and the SPC team nurse. Each patient kept the tablet throughout the study period or until the terminal stage of illness occurred. Video consultations were initiated by 1 SPC nurse. Video calls were encrypted to secure confidential information. An app was developed for relatives to participate in group consultations. Supervision was available from the company supplying the tablets. A total of 7 tablets were available during the study (Figure 1).

### Participants

In this study, 1 SPC team nurse recruited the patients.

The inclusion criteria include the following: patients with need for SPC, aged above 18 years, who agreed to participate and were willing to employ the tablet. Relatives were asked for participation when they occurred in the video consultations.

The exclusion criteria were patients who were too cognitively impaired to enter on their own and relatives who did not participate in video consultations.

Location, age, sex, diagnosis, relatives, and reason(s) for contacting the SPC team professionals were described to illuminate which patients and relatives were suitable for video consultations and how these took place.

### Ethical Approval

Informed consent was obtained from all individual participants included in the study. The study is registered at the Danish Data Protection Agency (file number: 2012-52-0018). The date of approval was September 29, 2016. This study was executed according to the Declaration of Helsinki [20].

### Data Collection

Participant observations were performed with regard to setting, behavior, and physical aspects, with inspiration from Spradley [21].

Interviews were carried out to gain firsthand information from patients and their relatives when participating in video consultations [22,23].

Participant observations were performed by one of the authors (KFF) during video consultations, directly followed by interviews, based on a semistructured interview guide (Multimedia Appendix 1).

The immediate interview made it possible to question and elaborate on the observed consultation. Image-based data were used to document the setting where participants used video consultations [24]. Method triangulation was used to validate and improve the understanding of participants’ experience in using video consultations [25].

### Data Analysis

Data analysis comprised 2 datasets: field notes from participant observations and interview transcripts. The observation guide and the interview guide were based on the research questions to create consistency. Interviews were audio recorded, transcribed ad verbatim, and all data were saved in a secure database, SharePoint.

The 2 datasets were thematically coded and categorized using NVivo-11 (QSR International), and a checklist for consolidated criteria for reporting qualitative research was used for reporting the study [26]. Interview transcripts were analyzed using the phenomenological descriptive approach described in Giorgi’s 4 steps [27-29]. The steps have been illustrated in Textbox 1. Examples are provided in Textbox 2.

Field notes from participant observations were thematically coded and compared with the interview transcripts. Main themes arising from the data analysis are described in the following section.

**The 4 steps in Giorgi’s descriptive analysis in a qualitative study about video consultations in specialized palliative care in Denmark.**

Step 1: To achieve an overall understanding, all transcribed material was read to reach the sense of the whole with maximum openness and by setting preunderstandings aside.

Step 2: When knowledge in the material was reached, a slower rereading was made to conduct meaning units. Text unrelated to the experiences in use of video consultations was deleted.

Step 3: A transformation of meaning units took place to create categories and concepts, which can express the meaning of interests from the researcher’s point of view.

Step 4: To investigate the meaning units based on the research questions, to reveal the essential structure, and to synthesize the transformed meaning units into descriptive statements.

**Examples of steps 2, 3, and 4 in the process of analysis as part of the main theme: video consultation strengthens communication despite technical difficulties.**

**Interview of Patient 3**

*Step 2: Meaning units described by patients*

- When you speak with them on the phone, you have this feeling that they turn up their nose at you, like you are some hypochondriac [...] but when you sit in front of the screen you see their facial expression, they listen to what you have to say.

*Step 3: Essential aspects described by researcher*

- The patient felt misunderstood during a telephone consultation as the facial expressions were left out.

*Step 4: Patients’ experiences*

- The use of the video consultation made the patient feel safe as the visualization of the specialized palliative care team nurse gave a trustworthy relation

## Results

### Overview

From September 2016 to March 2017, 14 participants were included. The study period was extended for 1 month as we wished to generate more descriptive data according to multiple video consultations in the study period. Furthermore, the included patients, who wished to continue to use video consultations in their SPC, were given the option to keep the tablet.

In this study, 11 patients with complex palliative care needs (79%) 7 men and 4 women, aged 30 to 68 years and 3 relatives (21%) were included. The total number of video consultations (n=86) varied from 3 to 18 per patient during the study period as the patients were included throughout the period and had different needs for video consultations with the SPC team nurse (Table 1).

A total of 3 main themes emerged from the descriptive data analysis:

1. Becoming an active patient in own care.
2. Video consultation strengthens communication, despite technical difficulties.
3. Gaining access for relatives.

**Table 1:** Patient (n=11) and relative (n=3) characteristics of Danish participants in an explorative study about video consultations in specialized palliative care.

Patient number and gender	Age, mean (n=59)	Diagnosis	Relative: Yes or No	Relatives included (n=3)	Video consultations (n=86) in the study period per patient (months)	Main topics in video consultation
Patient 1, male	64	Head and neck cancer	Yes	1 spouse, 1 daughter	13 October 2016 to March 2017 (6 months)	Pain relief
Patient 2, female	65	Bile duct cancer	No	—^a^	11 October 2016 to March 2017 (6 months)	Pain relief and nausea
Patient 3, male	63	Rectal cancer	No	—	18 November 2016 to March 2017 (5 months)	Pain relief
Patient 4, male	68	Prostate cancer	Yes	—	7 November 2016 to February 2017 (4 months)	Pain relief and dizziness
Patient 5, male	60	Prostate cancer	Yes	—	4 November 2016 to December 2016 (1 month)	Pain relief
Patient 6, female	64	Ovarian cancer	Yes	—	4 November 2016 to December 2016 (1 month)	Insomnia
Patient 7, male	30	Cystic fibrosis	Yes	—	9 January 2017 to March 2017 (3 months)	Nausea
Patient 8, female	67	Head and neck cancer	No	—	6 February 2017 to March 2017 (2 months)	Increased saliva
Patient 9, female	66	Lung cancer	No	—	6 January 2017 to March 2017 (2 months)	Pain relief and psychosocial
Patient 10, male	59	Lung cancer	Yes	1 spouse	3 February 2017 to March 2017 (2 months)	Dyspnea
Patient 11, male	47	Thymus cancer	No	—	5 February 2017 to March 2017 (2 months)	Pain relief

^a^No relatives participated.

**Table 2:** Cause of exclusion to participate in video consultations in specialized palliative care in Denmark.

Cause of exclusion	Number of patients (n=18), n (%)
Cognitive impairment due to illness progression	3 (17)
High risk of death before startup	3 (17)
No use of video consultation due to hospital admission	1 (6)
No tablets available	4 (22)
Lack of electronic skills	2 (11)
Suicidal patient	1 (6)
Hearing impairment	1 (6)
No desire for video consultations	3 (17)

### Becoming an Active Patient in Own Care

A majority of the patients were familiar with using a tablet from their everyday lives.

In the study, the patients found the tablet easy to use, following an introduction:

It´s so easy to use, it´s like she´s sitting right in front of you[...]I really think this is the future.

Patient 4

**Figure 2:**
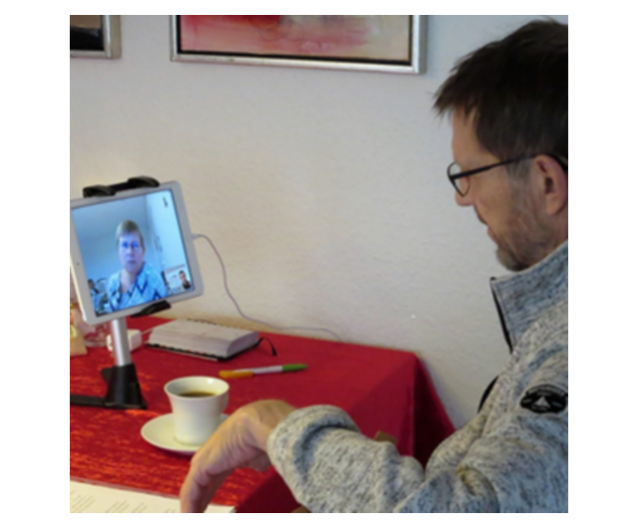
Patient using video consultations in specialized palliative home care.

They were pleased that they could see the SPC team nurse on the screen during the video consultations when discussing different issues (Figure 2).

During the participant observations, it was notable that patients were prepared for upcoming video consultations as questions were written down or discussed with the community nurses or relatives:

The tablet is placed on the dining table, so the patient’s wife and the community can see the screen. Notes were written for questions.

Patient 5

Although patients found the use of video consultations feasible in their contact to the SPC team nurse, they did not agree on when to start using the video consultation in relation to their physical conditions. Moreover, 1 patient found the use of video consultation more intense than using the telephone for consultations and did not find its use feasible later in her illness progression:

I don´t think the tablet will help me later on as I´m getting worse. At that time, it will be the community nurses who take over because I will have more physical needs. Some things cannot be taken care of on a tablet.

Patient 6

Another patient found telephone consultations more convenient in relation to how her condition was at the time but figured that visual contact would be more beneficial for the SPC team nurse later on:

Right now, I could just as easily talk to her (the SPC team nurse) on the phone as I could talk to her using video[...]Perhaps if I felt worse it[the video consultations] would be beneficial[for the SPC team nurse] as she would be able to consider my situation.

Patient 2

### Video Consultation Strengthens Communication, Despite Technical Difficulties

A total of 3 patients were observed experiencing minor problems finding the correct button for activating the tablet or controlling the volume button:

The patient seems insecure in how to start up the tablet but comfortable enough to press all the buttons...

Patient 8

They kept pressing different buttons until the right screen appeared or the volume was suitable; however, they did not express any difficulties when using the tablet during interviews:

This is great, I have to say[...] there´s no problem with this.

Patient 8

A total of 2 patients with limited ability to speak up owing to tracheotomy experienced great support using the visual communication:

She sees the colors on my face or when I´m tired or feeling bad.

Patient 1

These patients were used to writing everything down when communicating.

The tablet gave patients an opportunity to see the person they spoke to, which made them feel safe. The use of telephone consultations was not unwelcoming, contrary to previous telephone consultations with other health care professionals, which had led to misunderstandings:

When you speak with them on the phone, you have this feeling that they turn up their nose at you, like you are some hypochondriac[...]but when you sit in front of the screen, you see their facial expression, and they listen to what you have to say.

Patient 3

Video consultation gave patients an option of a clearer communication with the SPC team nurse because it generated the possibility to ask questions if anything was unclear or misunderstood as facial expressions showed confusion, etc.

Moreover, 1 patient mentioned the number of participants could affect the video consultation if too many joined the consultation:

When so many have to join in [the video consultation], you need to know when to be quiet when someone wants to say something[...]which would be more obvious if we sat in front of each other.

Patient 10

If multiple participants from the SPC team are attending the video consultation, it must be clear who is speaking so that misunderstandings and interruptions can be avoided.

In 4 cases, out of the 11 participant observations, an unclear image or reduced volume occurred; however, it did not seem to influence the conversations between patients and the SPC team nurse. The tablet showed the image of the SPC team nurse and then they had the consultation by telephone. The patients stated that it was satisfying to have scheduled appointments because it made it easier to plan other things and for relatives who lived in other parts of the country to participate:

We have set a date and that´s fine by me

Patient 4

### Gaining Access for Relatives

Relatives could join video consultations on equal terms with patients. They attended the consultation with the patients at home or from a distance, using an app, which gave them the ability to ask the SPC team nurse about treatments, appropriate times for visiting, etc.

Visualization had great value among relatives who played an active role in medical administration or scheduling appointments with the doctor:

I think it´s all right, if something is missing because then I can elaborate.

Relative 3

This opportunity to share the video consultation with a relative was found comforting to patients as no one had to make follow-up phone calls if anyone missed out:

Well, the names of the pills, forget it! I can´t remember any of them. She[relative]knows what I take and don’t take. I feel good about that and I appreciate that she´s there.

Patient 11

Furthermore, one relative joining a video consultation through the app on a cell phone was excited about the opportunity it provided. Due to living and working in another part of the country, the app made it possible to join in, which would not be possible over telephone:

Especially when he[patient 1] has the type of cancer that makes it difficult to speak. It has a great impact that you can see each other.

Relative 1

## Discussion

### Principal Findings

The study describes experiences of using video consultations among patients and their relatives in SPC at home. Both patients and relatives found visualization of the SPC team nurse comforting; however, if too many participants joined in, it could negatively affect consultations. Different perspectives were described on when to initiate the video consultations. Finally, relatives could participate more actively, which gave patients a sense of security as relatives helped with medication or elaborating on medical conditions.

### Embracing the Technology

During this study, patients referred to the use of tablets for video consultations as easy, convenient, and recognizable. This embodied relation, according to a post phenomenological approach as described by Don Ihde, occurs when a device, such as the tablet in use for video consultations, becomes incorporated in people’s everyday lives [30].

A transformation of roles was identified as video consultations enabled visual communication between participants, which gave patients a sense of security as the SPC team nurse appeared on the tablet screen. The technology-mediated contact made it possible for all participants to be active in a dialogue instead of being listeners or observers.

A total of 3 patients were observed experiencing minor difficulties activating the tablet; however, they did not express any issues with the item during the interview. This might be caused by awareness of being part of a study carried out by the organization providing their palliative care and due to the researchers’ overt roles in their homes [31,32].

The need to use video consultations differed. One patient found it useful at the time whereas another predicted it becoming more convenient as the illness progressed. Stern et al found the use of telecare-like video consultations viable, recommending such intervention early in palliative care. Due to illness progression and the feeling of being overwhelmed by the technology, early introduction was found beneficial for the patients and their relatives [9]. According to van Gurp et al, long-term interaction during teleconsultations results in trusting relationships and a feeling of relief and intimacy [18]. According to Huniche and Olesen, the constitution of health care changes when technology is adopted. The use of tablets constitutes a new method in SPC, and a technologized mediation invites particular ways of taking action [33]. This was shown in this study; the patients’ roles were pulled in the direction of cooperation and away from traditional care because they were taking an active role when preparing for video consultations. The patient-relative cooperation was strengthened as relatives could join in, strengthening a supporting role as they could elaborate when patients consulted the SPC team nurse.

### Will the Video Consultations Embrace the Palliative Needs?

According to WHO, palliative care should implicate psychosocial and spiritual problems as well as physical problems to relieve suffering [1]. The use of technology can improve health care access and create cost savings. In addition, the chronically ill and elderly may receive care while remaining at home [34]. However, the question is whether WHO’s recommendations for palliative care can be met using video consultation [1,5,34]. According to the European Association of Palliative Care, good communication is essential to palliative care, and sometimes painful concerns have to be considered, which requires time and commitment [35]. In this study, the issues being discussed were mainly medical adjustments due to pain relief (as shown in Table 1) rather than considerations of sensitive subjects widely, which might occur in the palliative care [35]. In addition to this, van Gurp et al showed that SPC team clinicians found the image of the patient a valuable and supportive addition, but they avoided talking about sensitive topics with vulnerable patients as they did not feel sufficiently close to be able to comfort them [18]. The patients in van Gurp et al’s study reported that physical distance provided exactly the freedom they needed to open up, after which normal life could be resumed [18].

This study showed that the simultaneous involvement of relatives in video consultations with the patient, or from a distance using an app, was both feasible and satisfying. This is in accordance with recommendations for palliative care, where relative involvement is recommended as the relatives suffer equally with the patient and after the patient has died [5].

### Strength and Limitations

The study was conducted as an explorative study, the first study in a Danish context conducted with patients’ and their relatives’ experiences during 82 video consultations in SPC at home. The SPC team nurse carried out the patient recruitment to avoid resistance to changes. When she was unavailable, patients were not included by other SPC team nurses. Patient inclusion was ongoing during the study period because of the focus on the increasing vulnerability of patients and their relatives. Some video consultations were rescheduled owing to hospice admission for optimizing stay.

The low participation from relatives (n=3) may be due to not all relatives being offered participation at the initial home visit.

Of the 18 excluded patients (Table 2), 7 were excluded owing to terminal stage of illness, cognitive impairment, or hospital admission caused by illness progression when first referred to the SPC. This could be prevented, according to Dalgaard, by early integration of palliative care, which may result in better symptom management, prolonged survival, improved patient perception of prognosis, and less aggressive care at the end of life [36].

The combination of participant observations and interviews helped vary the study and express discrepancies during fieldwork [37]. The phenomenological approach was helpful in maintaining openness as well as setting preconceptions aside in the process when exploring the participants’ experiences in using video consultations in SPC at home [29].

The findings in this study will be accompanied by a study focusing on health care professionals, the feasibility of video consultations in their work, and the organizational changes that may occur owing to implementing video consultations in the SPC team care.

### Clinical Implications and Future Research

As video consultations seem feasible and the study was initiated from 1 SPC team, it could be relevant to explore the feasibility in more SPC teams in an international context [38]. The analysis of qualitative data could be hypothesis-generating, with the aim of conducting a quantitative study. In this study, the qualitative data could generate the initial stages in identifying issues for respondents in questionnaire development [39]. Caring for the relatives in SPC is recommended [5], and by involving a family focusing approach in the video consultations, the patients and relatives can contribute with knowledge and experiences from their everyday lives [40].

### Conclusions

The use of video consultations strengthens communication. Despite their serious illness, patients took an active role, and despite technical issues, the tablet allowed access for the relatives, both present and via app, to attend in equal terms as the patient in the video consultation. The video consultations require introduction and reliable internet access before the video consultation can take place. Furthermore, agreement must be sought in SPC at home before video consultations are initiated and for as long as they continue.

## Appendix

Multimedia Appendix 1 Observation guide and semistructured interview guide in an explorative study about video consultations in specialized palliative home care in Denmark.

## Abbreviations

SPC: specialized palliative care
WHO: World Health Organization
