# Supplementary material for: Experiences With Video Consultations in Specialized Palliative Home-Care: Qualitative Study of Patient and Relative Perspectives
Source: J Med Internet Res. 2019 Mar 21;21(3):e10208. doi: 10.2196/10208 (PMC6447990; doi:10.2196/10208)
Supplement: Multimedia Appendix 1 [file jmir_v21i3e10208_app1.pdf]

Multimedia Appendix 1: Observation guide and semi-structured interview guide in an explorative study about video-consultations in specialized palliative home care in Denmark.

**Observation-guide**<br>The people:<br>How do the patient and/relative handle the tablet?<br>Is it easy to use/?<br>Do they (patient/relative) say anything about the tablet?<br>Is it difficult to use?<br>Physical setting:<br>Where does the video consultation take place? Bedroom? Livingroom?<br>Behavior:<br>Behavior: before, during and after the video consultation?<br>Transitions in behavior: before, during and after the video consultation?<br>Relation to SPC team nurse:<br>Eye-contact during use?<br>Can the patient see the SPC team nurse?<br>Verbal / nonverbal communication?
**Semi-structured interview-guide**<br>1: Using the technology<br>*“How do you find using a tablet when speaking to the SPC team nurse?”*<br>*“How do you find it?”*<br><br>2: Experiences of palliative care when it takes place via a screen<br>*“How do you experience your treatment when you´re using video consultation?”*<br>*“Compared to your other hospital contacts or visits, or telephone consultations?”*<br><br>3: Communication through a screen<br>*“What does it mean to you that you can see and hear the SPC team nurse?”*<br><br>4: The transformation of the palliative care, does it change when using video?<br>*”What did you get out of the consultation with the SPC team nurse?”*
